# P-231. Healthcare Utilization and Costs in People Living with HIV and Cardiovascular Disease in the United States

**DOI:** 10.1093/ofid/ofaf695.453

**Published:** 2026-01-11

**Authors:** Sean P Fleming, Shweta Kamat, Girish Prajapati, Viktor Chirikov, Wenying Quan, Mark Bounthavong

**Affiliations:** Merck & Co., Inc., Rahway, NJ, USA, Philadelphia, Pennsylvania; OPEN Health Evidence & Access, New York, New York; Merck & Co., Inc., Rahway, NJ; OPEN Health Evidence & Access, New York, New York; OPEN Health Evidence & Access, New York, New York; University of California, San Diego, La Jolla, California

## Abstract

**Background:**

Aging people living with HIV (PLWH) have higher prevalence and increased risk of comorbidities such as cardiovascular disease (CVD). This study assessed incremental all-cause healthcare resource utilization (HCRU) and costs among PLWH with and without CVD in the US.

**Figure:**
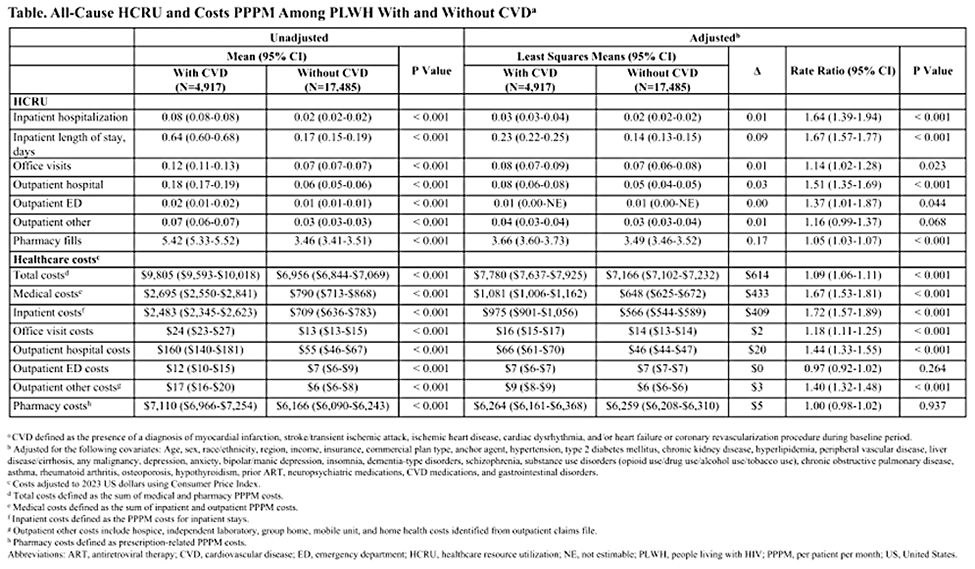

**Methods:**

A retrospective analysis of US administrative claims (Jan 2020-Dec 2022, Optum’s de-identified Clinformatics® Data Mart Database) examined all-cause HCRU and costs among adult (≥ 18 years) PLWH with ≥ 1 pharmacy claim for anchor antiretroviral therapy (ART) agent (NNRTI, PI, or INSTI) in 2021 (index date: earliest anchor ART claim). PLWH were followed to the earliest of 12 months or end of continuous enrollment and stratified into 2 groups based on the presence of CVD (yes/no) during baseline (12 months pre-index) using *ICD-10* diagnosis codes from medical claims. Multivariable generalized linear models with negative binomial/Poisson distribution (HCRU) and gamma distribution (costs) estimated differences in all-cause per-patient-per-month (PPPM) HCRU and costs (adjusted to 2023 USD) between groups, adjusting for baseline characteristics.

**Results:**

Of 22,402 PLWH identified, 4,917 (22%) had CVD. PLWH with vs. without CVD were older (mean age 61.45 vs. 52.86 years), more were women (22% vs. 18%) and Black (32% vs. 30%), and had higher mean Quan-Charlson Comorbidity Index scores (2.85 vs. 0.87) and baseline total costs ($5,574 vs. $3,442); all p < 0.001. Unadjusted all-cause PPPM HCRU and costs were significantly higher in PLWH with vs. without CVD (all p < 0.001; Table). In multivariable analyses, PLWH with vs. without CVD had significantly greater all-cause PPPM HCRU, total costs (9% higher), medical costs (67% higher), and inpatient costs (72.3% higher) (all p < 0.001; Table).

**Conclusion:**

PLWH with CVD experience a greater HCRU and cost burden than those without CVD. Identifying modifiable CVD risk factors (e.g., hypertension, type 2 diabetes mellitus) along with providing individualized HIV care might mitigate increases in HCRU and costs while optimizing care for PLWH.

**Disclosures:**

Sean P. Fleming, PhD, MSW, Merck & Co., Inc., Rahway, NJ, USA: Employee|Merck & Co., Inc., Rahway, NJ, USA: Stocks/Bonds (Public Company) Shweta Kamat, MS, PhD, Merck & Co., Inc., Rahway, NJ, USA: Contracted research Girish Prajapati, M.B.B.S., MPH , Merck & Co., Inc.: Employee|Merck & Co., Inc.: Stocks/Bonds (Private Company) Viktor Chirikov, MS, PhD, Merck & Co., Inc., Rahway, NJ, USA: Contracted research Wenying Quan, MS, Merck & Co., Inc., Rahway, NJ, USA: Contracted research Mark Bounthavong, PharmD, PhD, Merck & Co., Inc., Rahway, NJ, USA: Consultant|University of California, San Diego: Employment

